# Biomimetic Fibrin Matrix Modulates Early Human Follicular Growth Dynamics in a Bioengineered Artificial Ovary Derived from Cryopreserved Tissue: In Vitro Morphometric Assessment

**DOI:** 10.3390/ijms27093799

**Published:** 2026-04-24

**Authors:** Mengyang Cao, Plamen Todorov, Cheng Pei, Gohar Rahimi, Christine Skala, Volodimir Isachenko

**Affiliations:** 1 Department of Obstetrics and Gynecology, Medical Faculty, Cologne University, 50931 Cologne, Germany; weimusjtu@gmail.com (M.C.); chengpei314@foxmail.com (C.P.); gohar.rahimi@amedes-group.com (G.R.); christine.skala@uk-koeln.de (C.S.); 2 Institute of Biology and Immunology of Reproduction, Bulgarian Academy of Sciences (BAS), 1113 Sofia, Bulgaria; plamen.ivf@gmail.com; 3 AMEDES Facharzt-Zentrum für Kinderwunsch, Pränatale Medizin, Endokrinologie und Osteologie GmbH, 90518 Cologne, Germany

**Keywords:** ovarian tissue cryopreservation, fibrin scaffolds, tissue-engineered ovary, follicle isolation

## Abstract

Ovarian tissue cryopreservation is the primary fertility preservation strategy for prepubertal girls and patients requiring urgent gonadotoxic therapy. However, the risk of reintroducing malignant cells has prompted the development of safer alternatives, including follicle isolation followed by three-dimensional scaffold encapsulation for transplantation. Fibrin is a promising biomaterial for bioengineered ovary construction, although its ability to support early human follicle maintenance remains unclear. Follicles isolated from cryopreserved ovarian tissues of six patients were encapsulated within fibrin scaffolds of graded concentrations (high, medium, low). After 7 days of in vitro culture, follicle survival and diameter change were quantified. A total of 282 follicles (45.4 ± 10.1 µm) were embedded into fibrin scaffolds. After culture, 237 viable follicles were detected, yielding an overall survival of 84%. Follicle diameter increased to 58.8 ± 12.0 µm. Follicle survival rates were comparable across groups, while mean follicle diameter was 56.3 ± 12.5 µm (high), 61.9 ± 13.4 µm (medium), and 57.4 ± 9.3 µm (low). Follicles cultured in medium-concentration fibrin demonstrated significantly larger diameters compared with both high and low groups (*p* < 0.05), with no difference between high and low groups. Fibrin-based bioprosthetic ovary scaffolds support short-term in vitro maintenance of isolated human follicles, preserving spherical morphology and granulosa cell layer integrity. Medium-concentration fibrin was associated with greater follicle diameter expansion compared with higher and lower concentrations, indicating that scaffold composition influences early morphometric changes during in vitro follicle culture.

## 1. Introduction

As cancer survival rates continue to rise, fertility preservation has become an essential component of long-term quality-of-life care for oncology patients. By 2020, more than 200 live births resulting from the transplantation of cryopreserved ovarian tissue had been reported, confirming the clinical feasibility of the method [1]. To prevent fertility loss caused by gonadotoxic therapies, ovarian cortical tissue can be surgically removed and cryopreserved before treatment is initiated and may later be thawed and transplanted to restore endocrine activity and reproductive function [2]. Following completion of cancer therapy, cryopreserved tissue fragments can be warmed and grafted to re-establish steroidogenic function and follicular activity [3]. Currently, ovarian tissue cryopreservation remains the only validated fertility preservation option for prepubertal girls and for women in whom treatment postponement is not clinically acceptable [4].

Nevertheless, tissue transplantation is contraindicated in malignancies associated with a high probability of ovarian metastasis, most notably leukemia, neuroblastoma, and Burkitt lymphoma. In such cases, grafting of whole ovarian tissue is linked to a substantial risk of malignant cell re-introduction and disease relapse [5]. To mitigate oncological risks, an alternative approach has been proposed, involving the isolation of follicles from cryopreserved ovarian cortex, followed by encapsulation into biocompatible three-dimensional (3D) scaffolds to generate transplantable bioengineered ovary constructs [6]. This system also offers a platform to investigate oocyte–somatic cell interactions, particularly the underexplored role of oocytes in modulating the physiology of surrounding follicular cells [7].

Scaffold systems for artificial ovaries have incorporated a wide range of natural and synthetic biomaterials designed to sustain follicle viability, morphology, redox balance, and dimensional growth both in vitro and post-transplantation. Among these, fibrin-based matrices have received particular attention due to translational biosafety, clinical injectability, physiological degradability, and mechanical adaptiveness controlled through fibrinogen–thrombin polymerization ratios [8]. Fibrin scaffolds are formed by thrombin-mediated enzymatic cleavage of fibrinogen, followed by biochemical cross-linking to establish a three-dimensional, semi-elastic mesh capable of entrapping cells in minimal volumes [9]. Network properties, including pore-density, elasticity, solute diffusion, and cellular anchorage capacity, are modulated by altering the concentrations of fibrinogen and thrombin, allowing scaffold microstructure to be tuned to follicular requirements [10,11,12].

Multiple studies in small animal models have demonstrated that fibrin-based ovary constructs can reconstitute estrous cyclicity, support follicular growth, enable ovulation, and lead to post-implantation embryo development. In mice, endocrine and reproductive functions have been restored after autotransplantation of fibrin-encapsulated ovarian units [13,14,15,16]. Notably, Kniazeva et al. demonstrated that fibrin-encapsulated primordial follicles restored estrous cycling in all infertile recipients, whereas successful pregnancies and live births were observed only when the hydrogel had been pre-loaded with vascular endothelial growth factor (VEGF) [16]. These findings highlight the importance of optimizing fibrin-based systems.

However, the scaffold composition found to be effective for murine follicles may be insufficient to support the structural and developmental demands of isolated human follicles. Specifically, fibrinogen–thrombin ratios that induce functional murine constructs yield scaffolds lacking adequate tensile resilience for sustaining purified human follicular units [17]. Consequently, the capacity of fibrin to sustain isolated human follicles remains a key translational knowledge gap. To date, only a limited number of studies have addressed this question, and the available evidence remains inconclusive. These processes may be influenced by several related factors, including cryoprotectant load, osmotic equilibration, and cytoskeletal sensitivity during thawing. In particular, controlled investigations examining follicle survival, growth dynamics, and diameter changes across different fibrin concentrations remain lacking [18,19].

Given the limited availability of cryopreserved human follicular material, systematic evaluation of follicular responses to graded fibrin microenvironments is essential for informing scaffold design. Therefore, in the present study, human follicles isolated from cryopreserved ovarian cortex fragments were cultured in fibrin scaffolds formulated at three polymer concentrations to assess short-term follicle maintenance and diameter expansion in vitro.

## 2. Results

**Figure 1:**
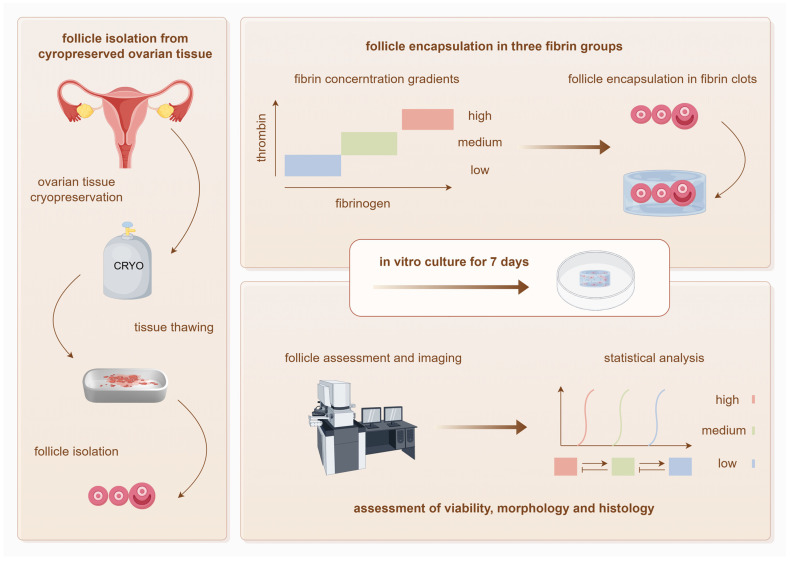
Schematic representation of the experimental workflow for evaluation of isolated follicle viability and dimensional development in graded fibrin-based tissue-engineered ovarian scaffold constructs.

The overall experimental workflow is illustrated in Figure 1.

**Figure 2:**
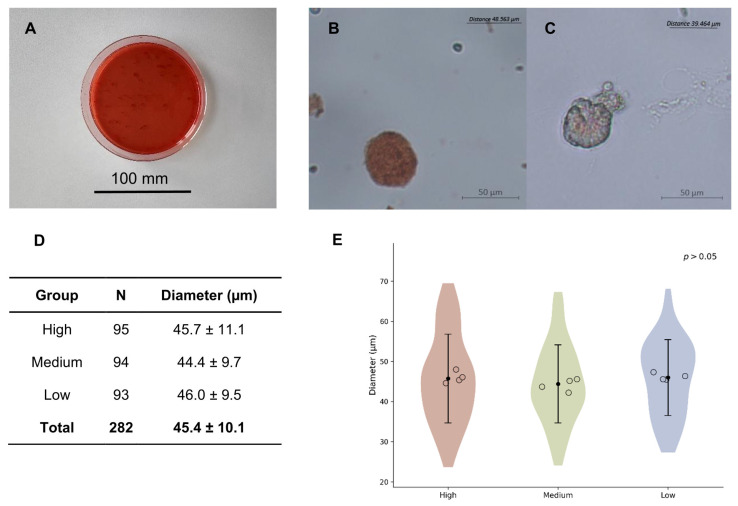
Morphological assessment of freshly isolated follicles. (**A**) Enzymatically digested ovarian tissue showing complete dissociation. (**B**) Representative bright-field images of a viable follicle stained with neutral red. (**C**) Representative image of a damaged follicle exhibiting weak or absent neutral red staining. (**D**) Total follicle numbers and mean diameters (mean ± SD) for each scaffold concentration. (**E**) Diameter distribution of isolated follicles prior to encapsulation. Violin plots represent the distribution of follicle diameters, while scatter points indicate mean values from independent experiments (*n* = 4). Due to limited follicle yield from individual donors, follicles from three donors were pooled in one experiment to ensure sufficient sample size, while the remaining experiments were conducted using single donors. No significant differences were observed among groups (one-way ANOVA, *p* > 0.05).

After thawing and enzymatic digestion, follicles that demonstrated intense neutral red staining and preserved a fully spherical morphology were classified as viable and aspirated for further processing (Figure 2). A total of 282 viable follicles were isolated, including 253 primordial follicles (<60 µm) and 29 primary follicles (60–75 µm). No secondary follicles (>75 µm) or other stage follicles were isolated. The initial mean follicular diameter was 45.4 ± 10.1 µm. Prior to encapsulation, follicles were washed to remove residual stromal cells and randomly allocated into three experimental groups: 95 follicles to the high-concentration fibrin scaffold group (mean diameter 45.7 ± 10.1 µm), 94 follicles to the medium-concentration group (mean diameter 44.4 ± 9.7 µm), and 93 follicles to the low-concentration group (mean diameter 46.0 ± 9.7 µm). Follicles at different developmental stages were evenly distributed across the three groups, with primary follicles numbering 12, 8, and 9 in the high-, medium-, and low-concentration groups, respectively. Variance homogeneity across the pre-encapsulation groups was confirmed using Levene’s test (*p* > 0.05). One-way analysis of variance (ANOVA) showed no statistically significant difference in mean diameters among these initial groups (*p* > 0.05).

**Figure 3:**
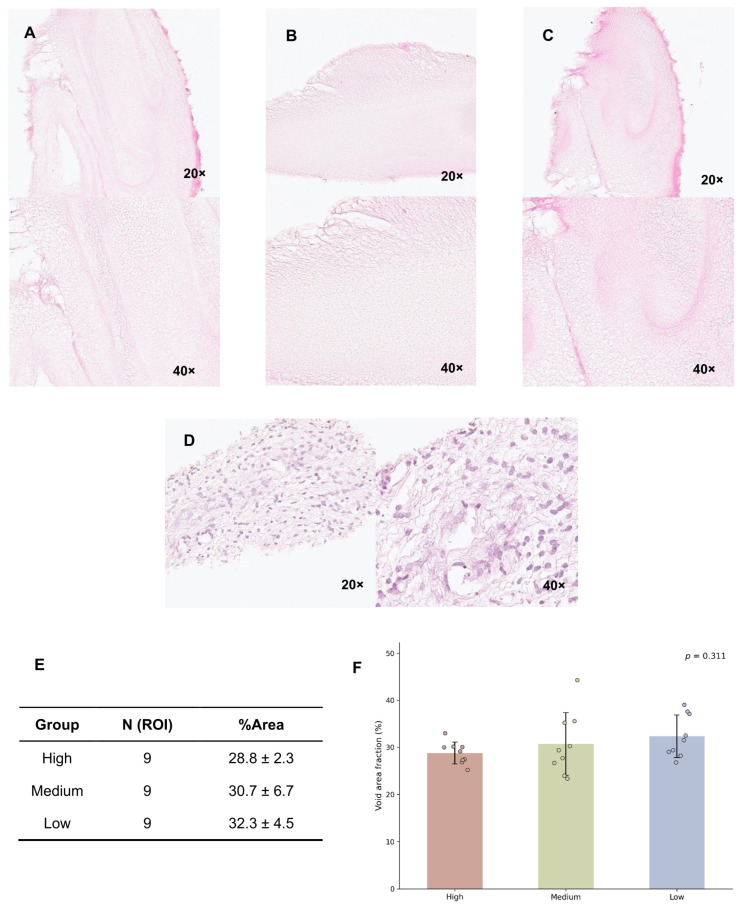
Histological evaluation of fibrin scaffolds and native ovarian tissue by hematoxylin and eosin (H&E) staining. Fibrin scaffolds of different concentrations exhibited comparable three-dimensional porous network structures composed of fibrin fibers under microscopic observation. (**A**) High-concentration fibrin scaffold; (**B**) medium-concentration fibrin scaffold; (**C**) low-concentration fibrin scaffold; (**D**) human ovarian tissue section. Magnification levels and scale were indicated in the lower right corners. (**E**) Void area fraction (mean ± SD) for each scaffold concentration. ROI, regions of interest. (**F**) Quantitative analysis of void area fraction in fibrin scaffolds. Bars represent mean ± SD, and scatter points represent individual ROI measurements. No significant differences were observed among groups (one-way ANOVA, *p* > 0.05).

Histological assessment of polymerized fibrin matrices prepared with Gunma University–compatible reagents revealed a porous fibrillar architecture with heterogeneously distributed voids. At the level of H&E staining, these structures appeared broadly comparable across fibrin concentrations and provided a structural framework potentially accommodating follicle embedding (Figure 3A–D). Quantitative analysis of H&E-stained sections showed that the void area fractions were 28.77 ± 2.34% in the high-concentration group, 30.70 ± 6.66% in the medium-concentration group, and 32.34 ± 4.52% in the low-concentration group (Figure 3E). Levene’s test indicated that the assumption of homogeneity of variances was satisfied (*p* > 0.05). One-way ANOVA revealed no statistically significant differences in void area fraction among groups (*p* > 0.05) (Figure 3F). Quantitative analysis of H&E-stained sections showed comparable void area fractions across fibrin concentrations, supporting the qualitative observation of similar porous architectures.

**Figure 4:**
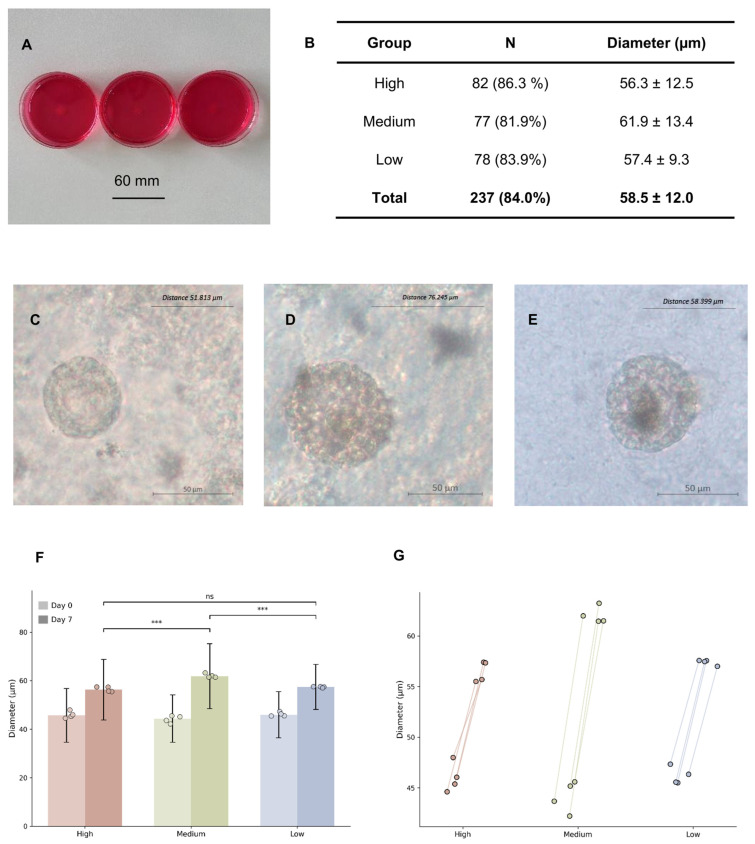
Morphological status and diameter changes of follicles after 7 days of in vitro culture in fibrin scaffolds of different concentrations. (**A**) Macroscopic appearance of fibrin scaffolds in the high-, medium-, and low-concentration groups. (**B**) Numbers of surviving follicles and mean follicle diameters (mean ± SD), with survival percentages indicated. (**C**–**E**) Representative bright-field images of viable follicles after culture in (**C**) high-, (**D**) medium-, and (**E**) low-concentration scaffolds. (**F**) Follicle diameter increased significantly after culture, with significant differences observed between the high- and medium-concentration groups and between the medium- and low-concentration groups (*** *p* < 0.001), while no significant difference was detected between the high- and low-concentration groups (ns, *p* = 0.77). (**G**) Experiment-level comparison of follicle diameter changes across fibrin scaffold concentrations (*n* = 4). Each point represents an independent experiment. Due to limited follicle yield from individual donors, follicles from three donors were pooled in one experiment to ensure sufficient sample size, while the remaining experiments were conducted using single donors.

Following 7 days of in vitro culture, follicle morphology and diameter were evaluated (Figure 4). Follicles maintaining spherical architecture with morphologically intact granulosa cell layers were categorized as viable, while disrupted or non-spherical structures were classified as damaged. Ultimately, 237 cultured follicles met the criteria for viability, corresponding to an overall survival rate of 84%. The final mean diameter increased to 58.8 ± 12.0 µm.

Post-culture survival rates were 86.3% (*n* = 82) for the high-concentration scaffold group, 81.9% (*n* = 77) for the medium-concentration group, and 83.9% (*n* = 78) for the low-concentration group. Mean diameters after culture were 56.3 ± 12.5 µm, 61.9 ± 13.4 µm, and 57.4 ± 9.3 µm, respectively (Figure 4E).

Levene’s test again confirmed homogeneity of variances among the post-culture diameter changes (*p* > 0.05). One-way ANOVA revealed a statistically significant difference in post-culture diameter changes among groups (*p* < 0.001). Tukey’s post hoc analysis indicated significant pairwise differences in post-culture diameter changes between the high- and medium-concentration fibrin scaffold conditions (*p* < 0.001) and between the medium- and low-concentration conditions (*p* < 0.001), whereas no significant difference was detected between high- and low-concentration scaffold groups (*p* = 0.77) (Figure 4F). To assess variability across independent experiments, experiment-level follicle diameter changes were visualized using scatter plots (Figure 4G). Despite moderate variability among experiments, a consistent pattern of greater diameter expansion in the medium-concentration group was observed.

## 3. Discussion

This study evaluated the ability of fibrin-based scaffolds to support short-term in vitro maintenance of isolated human follicles. Within the clinical context of ovarian tissue cryopreservation and the need to avoid malignant cell reintroduction, scaffold-based follicle reconstruction has emerged as a promising strategy. Although fibrin has been widely used in bioengineered ovary systems, its capacity to support early-stage human follicles remains incompletely characterized. In this study, an intermediate fibrin concentration was associated with improved early follicle growth compared with higher and lower concentrations.

In this study, fibrin scaffolds supported short-term survival (84%) and measurable diameter expansion of isolated human follicles after 7 days of in vitro culture. These findings indicate that fibrin provides a permissive microenvironment for early follicular maintenance within physiologically tolerable conditions. Previous studies have suggested that fibrin concentration critically influences scaffold performance, with low concentrations leading to unstable polymerization and high concentrations restricting nutrient diffusion. Consistent with these observations, our results demonstrate that intermediate fibrin conditions are more favorable for early follicle growth. These considerations informed the design of the present three-level concentration system.

After culture, survival rates were comparable across fibrin concentrations, whereas follicles in the intermediate group exhibited greater diameter expansion than those in the high and low groups. Statistical analyses were primarily performed at the follicle level due to the limited availability of human ovarian tissue and the variable follicle yield obtained from individual donors. However, follicles derived from the same donor are not fully independent biological replicates, which may introduce pseudo-replication. To partially address this issue, donor-level variability was visualized using scatter plots. Although some variability was observed, the medium-concentration group generally showed greater diameter expansion compared with the high- and low-concentration groups. These findings show that an intermediate fibrin concentration—approximately half-stock fibrinogen–thrombin formulation—is associated with greater early follicle diameter expansion and may represent a favorable microenvironment during short-term in vitro culture. Moreover, these results also suggest that scaffold composition plays a critical role in shaping the microenvironment that governs early follicular responses.

Quantitative analysis demonstrated comparable void area fractions across fibrin concentrations, indicating similar gross porosity. This may partially explain the comparable follicle survival rates, as nutrient diffusion is likely maintained under these conditions. However, the observed differences in follicle growth suggest that factors beyond bulk porosity, such as microstructural organization or mechanical properties, may play a more critical role. In addition, the present analysis was based on two-dimensional sections, which may not fully capture the three-dimensional architecture of the scaffold. To achieve more rigorous structural characterization and better approximate the native ovarian microenvironment, future studies should incorporate scanning electron microscopy (SEM) and transmission electron microscopy (TEM) to quantify scaffold porosity and fibrin fiber dimensions at the nanoscale.

To date, only a limited number of studies have evaluated the capacity of fibrin-based scaffolds to support the in vitro growth of isolated human follicles. In a representative study, Chiti et al. systematically examined scaffold architecture, porosity, and mechanical stiffness across a range of fibrinogen/thrombin (F/T) concentration ratios and demonstrated that the F50/T50 formulation most closely recapitulated both the ultrastructure and cortical rigidity of the human ovarian cortex [19]. These observations are consistent with the findings of the present study. Accordingly, the observed differences across fibrin concentrations may be related to concentration-dependent changes in scaffold properties, such as porosity, fibril architecture, mechanical stability, and degradation dynamics. These factors may influence the balance between hydration, nutrient diffusion, and structural support, thereby affecting follicle morphology and expansion. At higher fibrin concentrations, increased fiber density and reduced pore size may restrict nutrient diffusion and limit the spatial expansion of encapsulated follicles. In contrast, lower fibrin concentrations may result in a mechanically weaker and more rapidly degradable matrix, which could compromise structural support and the stability of the follicular microenvironment [9,20,21,22]. An intermediate fibrin concentration may therefore provide a more balanced microenvironment, permitting sufficient nutrient exchange while maintaining adequate mechanical integrity to support follicle morphology and early expansion. Nevertheless, the present study did not directly quantify scaffold ultrastructure or mechanical properties, and the proposed mechanisms remain inferential. Future investigations incorporating rheological measurements, high-resolution imaging, and molecular readouts will be necessary to more precisely define the relationship between fibrin scaffold properties and follicular responses.

In this study, a 7-day culture period was selected as an early assessment window to evaluate follicle survival and structural integrity following encapsulation, which are critical parameters for validating scaffold performance. Because encapsulated follicles are grafted without a pre-existing vascular network, they are exposed to a transient ischemic phase until revascularization is established. Transplantation studies using the chick chorioallantoic membrane and severe combined immunodeficient (SCID) mouse models have demonstrated that neovascularization of ovarian tissue grafts is typically initiated within the first week and achieves sufficient oxygenation and nutrient delivery by approximately 10 days [23,24,25]. Importantly, the greatest follicular loss has been reported to occur during this early post-graft period [26,27]. Accordingly, this time frame is widely used in follicle culture studies to assess early follicle viability and matrix biocompatibility both in vitro and in vivo [13,15,18,28,29].

The ultimate goal of human artificial ovary technology is to restore both endocrine and reproductive function following transplantation, comparable to native ovarian tissue. Although several prototypes have demonstrated short-term support of follicle survival and early development (within 10 days) in both murine and human models, long-term functional outcomes—such as ovulation, steroid hormone production, and the generation of healthy embryos and offspring—remain largely unexplored. To date, the longest reported transplantation period for a human bioprosthetic ovary is five months, as reported by Dolmans et al.; however, no evidence of functional ovarian activity, including hormone production, ovulation, or offspring generation, was observed during this period [30]. Taken together, these findings highlight the limited availability of long-term functional data for human artificial ovary transplantation, which may reflect constraints related to tissue availability and ethical considerations. Accordingly, the absence of long-term culture and transplantation data represents a limitation of the present study and will be a key focus of future investigations to better understand matrix–cell interactions and long-term graft functionality.

Furthermore, the experiments were carried out entirely in vitro, a setting that cannot fully reflect the physiological and metabolic environment encountered by follicles in vivo. Therefore, in vivo analyses assessing follicular dynamics after encapsulation into optimized hydrogels and subsequent grafting are considered essential. Analyzing follicle survival, migration behavior, endocrine recovery, and developmental trajectories following transplantation is proposed as the next research phase to expand translational relevance and reinforce clinical applicability of fibrin-based bioprosthetic ovary systems [31].

Fibrin is widely used in tissue engineering due to its high biocompatibility, low immunogenicity, and clinical applicability. In addition, fibrin can incorporate and release bioactive macromolecules, thereby enabling tailored biological functions such as angiogenesis and soft tissue regeneration [32,33]. Fibrin is also highly compatible with a wide range of natural and synthetic polymers, enabling the fabrication of composite matrices tailored to specific application requirements [20,21]. However, the high degradability and low rigidity remain significant drawbacks of fibrin-based scaffolds, which often lead to insufficient structural support before adequate cell proliferation and tissue regeneration [20,21,34]. One potential strategy is to combine fibrin with other natural or synthetic polymers to create composite matrices that enhance mechanical strength and reduce degradation.

Beyond fibrin, additional natural (e.g., alginate), synthetic (e.g., Polyethylene Glycol (PEG)), and tissue-derived matrices, including decellularized extracellular matrix (dECM), have been used for ovarian bioengineering [35,36,37,38]. Because alginate is poorly degradable, alginate–fibrin composite matrices have been widely applied in tissue engineering, for example, for encapsulating stem cells to promote bone regeneration. However, follicle survival and growth within alginate–fibrin composites have not yet been reported. Therefore, this strategy may represent a promising and feasible direction for the design of artificial ovaries [22,39,40]. PEGylation of fibrin has been shown to increase mechanical stiffness and reduce fibrinolytic degradation, improving structural stability against proteolysis [41,42]. Importantly, two independent human studies by Dadashzadeh et al. indicated that PEGylated fibrin hydrogels, when prepared at optimized ratios, supported stromal ovarian cell viability and proliferation in vitro, suggesting translational potential for human follicle encapsulation [43,44]. To better compare the properties of fibrin relative to alternative biomaterials used in artificial ovary construction, a comparative summary of commonly used scaffold materials is provided in Table 1.

Collectively, the present study demonstrates that fibrin provides a supportive platform for short-term in vitro maintenance of isolated human follicles, with scaffold composition influencing early morphometric outcomes. Future studies integrating mechanical characterization, molecular analyses, and long-term functional assessments will be necessary to elucidate matrix–cell interactions and validate these observations.

## 4. Materials and Methods

### 4.1. Ethics and Experimental Design

Human ovarian tissue was obtained with written, informed consent from patients undergoing laparoscopic surgery in the Department of Gynecology and Obstetrics, University Hospital of Cologne. The use of the human ovarian tissue was approved by the Clinical Ethics Committee of University Hospital of Cologne (applications 999184 and 13-147). Ovarian cortical biopsies were collected from six patients aged 17–35 years, whose underlying clinical diagnoses included hematologic malignancies (*n* = 3), brain tumors (*n* = 2), and breast carcinoma (*n* = 1). Table 2 summarizes the basic demographic and clinical characteristics of donors.

Follicles were isolated from previously cryopreserved ovarian cortical fragments and encapsulated within fibrin scaffolds prepared at high, medium, and low fibrinogen concentrations. Constructs were cultured in vitro for 7 days, after which follicle viability (survival rate, %) and diameter changes (µm) were measured and recorded for statistical analyses. All experimental culture cycles were performed independently on four separate experimental days to ensure biological reproducibility. In three experiments, follicles were isolated from individual donors. In one experiment, due to limited follicle yield, tissues from three donors were pooled prior to follicle isolation to ensure sufficient sample size for downstream analysis. A portion of the tissue samples was also used for histological analysis.

### 4.2. Freezing of Ovarian Tissue

Freezing of ovarian tissue was performed in accordance with previously published protocols [45,46]. Ovarian cortical tissue fragments were incubated for 30 min at controlled laboratory ambient temperature in 20 mL of cryopreservation medium composed of basal buffer supplemented with 6% DMSO, 6% ethylene glycol, and 0.15 mol/L sucrose. After equilibration, the fragments were transferred into sterile 5 mL cryovials prefilled with 4.5 mL of the same cryopreservation medium.

Controlled slow cooling was applied using the SyLab IceCube 14S programmable freezer (Neupurkersdorf, Austria). Ice nucleation was initiated through automated seeding at −6 °C. The samples were then cooled from −6 °C to −34 °C at −0.3 °C/min to enable gradual CPAs permeation and cellular dehydration while limiting intracellular ice formation. At −34 °C, cryovials were directly plunged into liquid nitrogen and stored until use.

### 4.3. Thawing of Ovarian Tissue

Ovarian tissue thawing was also carried out following the procedures previously published by our group [45,46]. Cryovials were retrieved from liquid nitrogen, exposed to ambient temperature for 30 s, and immersed in a boiling water bath (100 °C) for 60 s until complete ice clearance was visually confirmed. Warming was terminated after the disappearance of macroscopic ice within the cryoprotective medium had been observed. The thawed ovarian fragments were immediately transferred into 10 mL of CPAs-dilution solution (basal buffer containing 0.5 M sucrose) in sterile 100 mL specimen containers obtained from Sarstedt (Nümbrecht, Germany). Stepwise removal of permeable cryoprotective agents was performed by continuous orbital agitation at 200 oscillations/min for 15 min at controlled ambient laboratory temperature.

### 4.4. Isolation of Fresh Follicles

Fresh follicles were isolated according to a previously published enzymatic dissociation protocol for human ovarian tissue [47,48]. After the removal of surrounding adipose tissue, ovarian cortical biopsies were rapidly fragmented using two sterile No. 22 micro-scalpel blades inside a 5 cm Petri dish under aseptic conditions to avoid prolonged ischemic exposure. The resulting fragments were immediately transferred into sterile centrifuge tubes prefilled with enzymatic dissociation solution composed of:

Liberase™ DH collagenase/thermolysin blend (0.28 Wünsch units/mL),
DNase I (10 µg/mL),
Dulbecco’s phosphate-buffered saline containing Mg^2+^ and Ca^2+^

at a scaling volume of 2 mL enzyme solution per 100 mg of initial ovarian tissue. Enzymatic dissociation was performed for 60 min at 37 °C using continuous orbital shaking at 130 rpm. Tissue fragments were homogenized by 1000 µL trituration every 30 min to increase digester penetration while limiting mechanical follicular collapse. Fifteen minutes prior to digestion completion, Neutral Red viability indicator was introduced at a final working concentration of 50 µg/mL to enable subsequent optical viability discrimination.

Warming digestion was terminated by the addition of an equal volume of ice-cold L 15 Medium supplemented with 20% fetal calf serum at 4 °C to avoid continued CPAs activity. Enzymatically liberated oocyte–cumulus complexes (EO-CCCs) with intact basement membranes were identified by stereomicroscopy and selectively retrieved using 125 µm V-polished glass capillary tips attached to Cook^®^ follicle-denuding micropipette handles under real-time oblique high-coherence contrast illumination optics to enhance surface topology and prevent misclassification of collapsed stromal aggregates.

### 4.5. Viability Assessment of Fresh Follicles

Follicles with dense crimson staining, preserved spherical morphology, and continuous basement membrane outlines were aspirated and classified as viable, whereas those with weak or absent staining or irregular membrane boundaries were categorized as structurally compromised. Follicle developmental stages were classified according to Gougeon and Fortune as follows: primordial follicles (<60 µm), oocytes surrounded by a single layer of flattened pre-granulosa cells; primary follicles (60–75 µm), oocytes with a single layer of cuboidal granulosa cells; and secondary follicles (75–200 µm), characterized by at least two complete layers of granulosa cells [49,50]. Isolated viable follicular units were assessed using the inverted Zeiss Axiovert 40 CFL microscope integrated with the ZEN imaging software (ZEN Blue™, Carl Zeiss AG, Jena, Germany) measurement module. Follicular diameters were calculated from the basement membrane perimeter using calibrated vector-distance evaluation tools within the “Graphics” layer, recorded to one decimal precision, and verified by dual-axis orthogonal diameter tracing to reduce membrane-flattening artifacts. Post-isolation, the follicular suspension was washed three times in chilled basal handling buffer to eliminate residual stromal and non-follicular somatic contaminants. The post-wash suspension was enriched by repeated microcapillary aspiration into Eppendorf tubes to minimize excess fluid volume and avoid fibrin-gel dilution prior to scaffold embedding.

### 4.6. Encapsulation of Isolated Follicles

Isolated human follicles were encapsulated using the clinical-grade fibrin matrix system Tisseel™, supplied as a dual-component formulation of purified human fibrinogen (91 mg/mL) and human thrombin (500 IU/mL). Scaffold concentration gradients were generated by diluting both components in sterile 0.9% NaCl saline buffer, yielding three experimental conditions optimized for differential network density: high concentration (68.3 mg/mL fibrinogen/50 IU/mL thrombin), medium concentration (45.5 mg/mL fibrinogen/25 IU/mL thrombin), low concentration (22.8 mg/mL fibrinogen/10 IU/mL thrombin).

**Figure 5:**
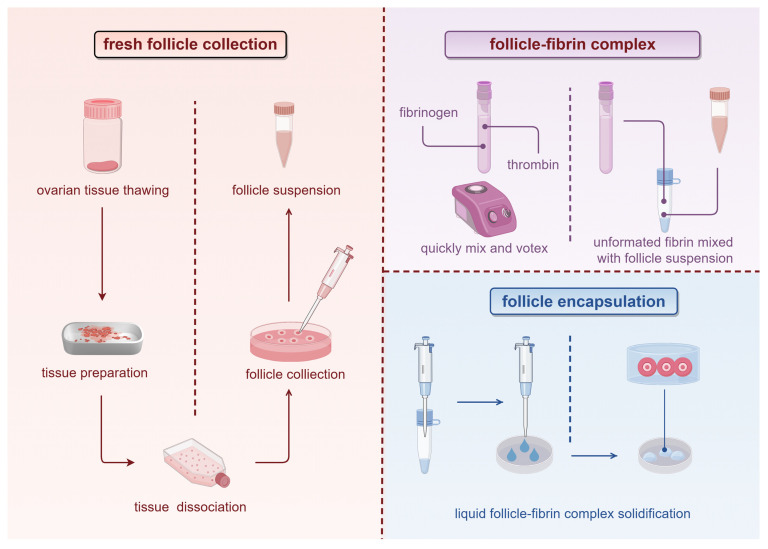
Construction of fibrin-based artificial ovary scaffolds from cryopreserved ovarian tissue. Cryopreserved ovarian tissue was first thawed and enzymatically processed to isolate viable follicles. After the follicle suspension was collected, fibrin precursor components (fibrinogen and thrombin) were prepared and mixed to form a liquid fibrin matrix. The follicle suspension was then combined with the fibrin solution for gelation, resulting in the encapsulation of follicles within a three-dimensional fibrin scaffold.

Encapsulation of isolated follicles was performed in accordance with previously published protocols [48]. All components were mixed in sterile Eppendorf microcentrifuge tubes and gently vortexed at low speed to ensure homogeneous distribution of the polymer precursor while preventing premature fibrin cross-linking prior to cell loading. The follicular suspension was then randomly assigned to the different fibrin formulations and added dropwise (20–30 µL per clot) to achieve uniform CPA-free encapsulation within the semi-solidifying fibrin matrix. After gelation, the constructs were transferred to high-optical-clarity imaging chambers (WillCo-dish™, WillCo Wells B.V., Amsterdam, The Netherlands) for microscopic assessment, thereby preserving the three-dimensional follicular architecture during polymer stabilization. Figure 5 summarizes the workflow to construct fibrin-based artificial ovary scaffolds from cryopreserved ovarian tissue.

### 4.7. In Vitro Culture of Encapsulated Follicles

Follicle-scaffold constructs were cultured in a standard humidified 37 °C incubator under 5% CO_2_ tension using α-MEM basal culture buffer (Gibco™, Thermo Fisher Scientific, Waltham, MA, USA), supplemented with fetal bovine serum (15%), L-glutamine (2 mmol/L), ITS metabolic support mix (Sigma™, Merck KGaA, Darmstadt, Germany), ascorbic acid (50 µg/mL), recombinant human FSH stimulation supplement (300 mIU/mL; Gonal-F™, Merck KGaA), penicillin-streptomycin antibiotic protection complex (100 IU/mL/0.1 mg/mL), and intracellular redox stabilizer α-lipoic acid (1 mM). For each experiment, ovarian tissue was thawed in the morning, and fresh follicles were isolated following enzymatic digestion for subsequent assessment and encapsulation. This day was defined as day 0 of culture. To minimize mechanical and temperature-induced stress, constructs were maintained in the incubator throughout culture and were only briefly removed every other day for medium exchange and morphological inspection under a stereomicroscope. Culture was terminated on day 8, and the fibrin constructs were retrieved for microscopic evaluation and follicle measurement.

Post-culture follicular integrity was assessed using bright-field inverted microscopy (Axiovert 40 CFL™, Carl Zeiss AG, Jena, Germany). Follicles retaining spherical structure, uninterrupted basement membrane outlines, and visibly preserved somatic corona layers were classified as viable. Follicular diameters were measured by calibrated vector-distance tracing from the basement membrane perimeter using built-in diameter measurement tools of stereomicroscopy acquisition software (ZEN Blue™, Carl Zeiss AG). Follicles exhibiting volume collapse, membrane deformation, substantial loss of cumulus or granulosa cell layers, or indeterminate 3D architecture inconsistent with spherical osmotic recovery were designated as structurally damaged.

### 4.8. Histological Analysis

Histological sections were stained with hematoxylin and eosin (H&E) following established laboratory protocols. Slides were deparaffinized in two consecutive immersions in 100% xylene (10 min each), rehydrated through a descending ethanol gradient (100%, 95%, 70%, 50%; 3–5 min per step), and rinsed in distilled water. Nuclear staining was performed using Mayer’s hematoxylin solution (1–3 min), followed by bluing in running tap water (3–5 min, adjusted to staining intensity). Cytoplasmic contrast counterstaining was conducted using eosin Y solution (~30 s). Stained slides were subsequently dehydrated through an ascending ethanol gradient (70%, 95%, 100%), cleared in two changes of xylene (1 min each), and permanently mounted under glass coverslips using a resin-based mounting medium. Digital slide acquisition was carried out with the Hamamatsu Slidescanner S360 (Hamamatsu, Japan) in bright-field mode. All histological images were annotated by NDP.view2 Image viewing software (NDP.view 2.9.22).

Image analysis was performed using ImageJ software version 1.54s (National Institutes of Health, Bethesda, MD, USA). For each experimental group (high, medium, and low fibrin concentrations), three independent sections were evaluated. From each section, three non-overlapping regions of interest (ROIs) were randomly selected at identical magnification to minimize sampling bias. All images were converted to 8-bit grayscale and subjected to thresholding to distinguish fibrin structures from void spaces. Threshold values were adjusted consistently across all images within the same analysis batch. The void area fraction (%) was calculated as the proportion of non-stained (void) regions relative to the total ROI area.

### 4.9. Statistical Analysis

Morphometric measurements of follicular diameter were recorded with single-decimal precision and summarized as mean ± standard deviation (SD). Group variance homogeneity was evaluated using Levene’s test. Diameter differences among experimental conditions (high, medium, low fibrin scaffold density) were tested by one-way analysis of variance (ANOVA). Pairwise mean comparisons were further resolved using Tukey’s multiple comparisons test. All statistical operations were executed in Python 3.13. A two-sided probability threshold of *p* < 0.05 defined statistical significance.

## 5. Conclusions

Fibrin-based bioprosthetic ovary scaffolds support short-term in vitro maintenance of isolated human follicles, preserving spherical morphology and granulosa cell layer integrity. Medium-concentration fibrin was associated with greater follicle diameter expansion compared with higher and lower concentrations, indicating that scaffold composition influences early morphometric changes during follicle culture.

## Figures and Tables

**Table 1:** Comparison of materials used for artificial ovary scaffolds.

Material	Advantages	Limitations	Matrix-Cell Interactions
fibrin	high biocompatibility; low immunogenicity; capable of incorporating bioactive factors; highly compatible with other polymers	low mechanical strength; rapid degradation; limited structural stability	supports early follicle development; enhances angiogenesis when incorporating bioactive factors
alginate	good structural stability; tunable stiffness; easy accessibility	poor biodegradability	lack of cell-binding sites; restrict follicle growth
PEG	easily manufactured; controlled degradation and mechanical properties	lack of intrinsic biocompatibility; potential adverse effects of degradation products	support proliferation of ovarian stromal cells
dECM	native tissue composition; provides natural biochemical factors	variability between samples; limited availability; complex preparation	mimics native ovarian microenvironment; supports follicle survival for longer time

PEG, Polyethylene glycol; dECM, Decellularized extracellular matrix.

**Table 2:** Basic demographic and clinical characteristics of donors.

Donor ID	Age (Years)	Diagnosis	Experimental Group
D1	25	breast carcinoma	experiment 1
D2	17	brain tumor	experiment 2
D3	35	brain tumor	experiment 3
D4	23	hematologic malignancy	experiment 4 (pooled)
D5	22	hematologic malignancy	experiment 4 (pooled)
D6	29	hematologic malignancy	experiment 4 (pooled)

## Data Availability

The original contributions presented in this study are included in the article. Further inquiries can be directed to the corresponding author.

## References

[1] Dolmans M.-M., Falcone T., Patrizio P. (2020). Importance of patient selection to analyze in vitro fertilization outcome with transplanted cryopreserved ovarian tissue. Fertil. Steril..

[2] Donnez J., Dolmans M.M. (2017). Fertility Preservation in Women. N. Engl. J. Med..

[3] Dolmans M.-M., Donnez J., Cacciottola L. (2021). Fertility Preservation: The Challenge of Freezing and Transplanting Ovarian Tissue. Trends Mol. Med..

[4] Rosendahl M., Andersen M.T., Ralfkiær E., Kjeldsen L., Andersen M.K., Andersen C.Y. (2010). Evidence of residual disease in cryopreserved ovarian cortex from female patients with leukemia. Fertil. Steril..

[5] Armstrong A.G., Kimler B.F., Smith B.M., Woodruff T.K., Pavone M.E., Duncan F.E. (2018). Ovarian tissue cryopreservation in young females through the Oncofertility Consortium’s National Physicians Cooperative. Future Oncol..

[6] Dolmans M.-M., Masciangelo R. (2021). Risk of transplanting malignant cells in cryopreserved ovarian tissue. Fertil. Preserv. Princ. Pract..

[7] Dolmans M.-M., Amorim C.A. (2019). Fertility preservation: Construction and use of artificial ovaries. Reproduction.

[8] Salama M., Woodruff T.K. (2019). From bench to bedside: Current developments and future possibilities of artificial human ovary to restore fertility. Acta Obstet. Gynecol. Scand..

[9] Cao M., Todorov P., Rahimi G., Salama M., Woodruff T.K., Isachenko E., Skala C., Isachenko V. (2025). Construction and Bioengineering of Human Bioprosthetic Ovaries from Cryopreserved Ovarian Tissue. Int. J. Mol. Sci..

[10] Tamadon A., Park K.-H., Kim Y.Y., Kang B.-C., Ku S.-Y. (2016). Efficient biomaterials for tissue engineering of female reproductive organs. Tissue Eng. Regen. Med..

[11] Peng X., Cheng C., Zhang X., He X., Liu Y. (2023). Design and application strategies of natural polymer biomaterials in artificial ovaries. Ann. Biomed. Eng..

[12] Shea L.D., Woodruff T.K., Shikanov A. (2014). Bioengineering the ovarian follicle microenvironment. Annu. Rev. Biomed. Eng..

[13] Luyckx V., Dolmans M.-M., Vanacker J., Legat C., Moya C.F., Donnez J., Amorim C.A. (2014). A new step toward the artificial ovary: Survival and proliferation of isolated murine follicles after autologous transplantation in a fibrin scaffold. Fertil. Steril..

[14] Smith R.M., Shikanov A., Kniazeva E., Ramadurai D., Woodruff T.K., Shea L.D. (2014). Fibrin-mediated delivery of an ovarian follicle pool in a mouse model of infertility. Tissue Eng. Part A.

[15] Chiti M.C., Dolmans M.-M., Orellana R., Soares M., Paulini F., Donnez J., Amorim C. (2016). Influence of follicle stage on artificial ovary outcome using fibrin as a matrix. Hum. Reprod..

[16] Kniazeva E., Hardy A., Boukaidi S., Woodruff T., Jeruss J., Shea L. (2015). Primordial follicle transplantation within designer biomaterial grafts produce live births in a mouse infertility model. Sci. Rep..

[17] Chiti M.C., Dolmans M.M., Lucci C.M., Paulini F., Donnez J., Amorim C.A. (2017). Further insights into the impact of mouse follicle stage on graft outcome in an artificial ovary environment. Mol. Hum. Reprod..

[18] Paulini F., Vilela J.M., Chiti M.C., Donnez J., Jadoul P., Dolmans M.-M., Amorim C.A. (2016). Survival and growth of human preantral follicles after cryopreservation of ovarian tissue, follicle isolation and short-term xenografting. Reprod. Biomed. Online.

[19] Chiti M.C., Dolmans M.M., Mortiaux L., Zhuge F., Ouni E., Shahri P.A.K., Van Ruymbeke E., Champagne S.D., Donnez J., Amorim C.A. (2018). A novel fibrin-based artificial ovary prototype resembling human ovarian tissue in terms of architecture and rigidity. J. Assist. Reprod. Genet..

[20] Brown A.C., Barker T.H. (2014). Fibrin-based biomaterials: Modulation of macroscopic properties through rational design at the molecular level. Acta Biomater..

[21] Chiti M.C., Dolmans M.M., Donnez J., Amorim C.A. (2017). Fibrin in Reproductive Tissue Engineering: A Review on Its Application as a Biomaterial for Fertility Preservation. Ann. Biomed. Eng..

[22] Dadashzadeh A., Moghassemi S., Shavandi A., Amorim C.A. (2021). A review on biomaterials for ovarian tissue engineering. Acta Biomater..

[23] Martinez-Madrid B., Donnez J., van Eyck A.-S., Veiga-Lopez A., Dolmans M.-M., van Langendonckt A. (2009). Chick embryo chorioallantoic membrane (CAM) model: A useful tool to study short-term transplantation of cryopreserved human ovarian tissue. Fertil. Steril..

[24] Van Eyck A.-S., Jordan B.F., Gallez B., Heilier J.-F., van Langendonckt A., Donnez J. (2009). Electron paramagnetic resonance as a tool to evaluate human ovarian tissue reoxygenation after xenografting. Fertil. Steril..

[25] Cacciottola L., Manavella D.D., Amorim C.A., Donnez J., Dolmans M.-M. (2018). In vivo characterization of metabolic activity and oxidative stress in grafted human ovarian tissue using microdialysis. Fertil. Steril..

[26] Roness H., Meirow D. (2019). FERTILITY PRESERVATION: Follicle reserve loss in ovarian tissue transplantation. Reproduction.

[27] Gavish Z., Spector I., Peer G., Schlatt S., Wistuba J., Roness H., Meirow D. (2018). Follicle activation is a significant and immediate cause of follicle loss after ovarian tissue transplantation. J. Assist. Reprod. Genet..

[28] Dolmans M.-M., Martinez-Madrid B., Gadisseux E., Guiot Y., Yuan W.Y., Torre A., Camboni A., van Langendonckt A., Donnez J. (2007). Short-term transplantation of isolated human ovarian follicles and cortical tissue into nude mice. Reproduction.

[29] Chiti M.C., Dolmans M.-M., Hobeika M., Cernogoraz A., Donnez J., Amorim C.A. (2017). A modified and tailored human follicle isolation procedure improves follicle recovery and survival. J. Ovarian Res..

[30] Dolmans M.-M., Yuan W.Y., Camboni A., Torre A., van Langendonckt A., Martinez-Madrid B., Donnez J. (2008). Development of antral follicles after xenografting of isolated small human preantral follicles. Reprod. Biomed. Online.

[31] Amorim C.A., Shikanov A. (2016). The artificial ovary: Current status and future perspectives. Future Oncol..

[32] Thomson K.S., Dupras S.K., Murry C.E., Scatena M., Regnier M. (2014). Proangiogenic microtemplated fibrin scaffolds containing aprotinin promote improved wound healing responses. Angiogenesis.

[33] Rajabzadeh A., Jahanpeyma F., Talebi A., Moradi F., Hamidieh A.A., Eimani H. (2020). Fibrin scaffold incorporating platelet lysate enhance follicle survival and angiogenesis in cryopreserved preantral follicle transplantation. Galen Med. J..

[34] Guthold M., Liu W., Sparks E., Jawerth L., Peng L., Falvo M., Superfine R., Hantgan R.R., Lord S.T. (2007). A comparison of the mechanical and structural properties of fibrin fibers with other protein fibers. Cell Biochem. Biophys..

[35] Vanacker J., Dolmans M.-M., Luyckx V., Donnez J., Amorim C.A. (2014). First transplantation of isolated murine follicles in alginate. Regen. Med..

[36] Rios P.D., Kniazeva E., Lee H.C., Xiao S., Oakes R.S., Saito E., Jeruss J.S., Shikanov A., Woodruff T.K., Shea L.D. (2018). Retrievable hydrogels for ovarian follicle transplantation and oocyte collection. Biotechnol. Bioeng..

[37] Kim J., Perez A.S., Claflin J., David A., Zhou H., Shikanov A. (2016). Synthetic hydrogel supports the function and regeneration of artificial ovarian tissue in mice. NPJ Regen. Med..

[38] Pors S., Ramløse M., Nikiforov D., Lundsgaard K., Cheng J., Andersen C.Y., Kristensen S. (2019). Initial steps in reconstruction of the human ovary: Survival of pre-antral stage follicles in a decellularized human ovarian scaffold. Hum. Reprod..

[39] Kreeger P.K., Deck J.W., Woodruff T.K., Shea L.D. (2006). The in vitro regulation of ovarian follicle development using alginate extracellular matrix gels. Biomaterials.

[40] Chen W., Zhou H., Weir M.D., Bao C., Xu H.H. (2012). Umbilical cord stem cells released from alginate–fibrin microbeads inside macroporous and biofunctionalized calcium phosphate cement for bone regeneration. Acta Biomater..

[41] Barker T.H., Fuller G.M., Klinger M.M., Feldman D., Hagood J. (2001). Modification of fibrinogen with poly (ethylene glycol) and its effects on fibrin clot characteristics. J. Biomed. Mater. Res. Off. J. Soc. Biomater. Jpn. Soc. Biomater. Aust. Soc. Biomater. Korean Soc. Biomater..

[42] Dikovsky D., Bianco-Peled H., Seliktar D. (2010). Proteolytically Degradable Photo-Polymerized Hydrogels Made From PEG–Fibrinogen Adducts. Adv. Eng. Mater..

[43] Dadashzadeh A., Moghassemi S., Amorim C.A. (2021). Evaluation of PEGylated fibrin as a three-dimensional biodegradable scaffold for ovarian tissue engineering. Mater. Today Chem..

[44] Dadashzadeh A., Moghassemi S., Peaucelle A., Lucci C.M., Amorim C.A. (2023). Mind the mechanical strength: Tailoring a 3D matrix to encapsulate isolated human preantral follicles. Hum. Reprod. Open.

[45] Isachenko V., Todorov P., Isachenko E., Rahimi G., Tchorbanov A., Mihaylova N., Manoylov I., Mallmann P., Merzenich M. (2015). Long-time cooling before cryopreservation decreased translocation of phosphatidylserine (Ptd-L-Ser) in human ovarian tissue. PLoS ONE.

[46] Isachenko V., Todorov P., Isachenko E., Rahimi G., Hanstein B., Salama M., Mallmann P., Tchorbanov A., Hardiman P., Getreu N. (2016). Cryopreservation and xenografting of human ovarian fragments: Medulla decreases the phosphatidylserine translocation rate. Reprod. Biol. Endocrinol..

[47] Chen J., Isachenko E., Wang W., Du X., Wang M., Rahimi G., Mallmann P., Isachenko V. (2022). Optimization of Follicle Isolation for Bioengineering of Human Artificial Ovary. Biopreserv. Biobank..

[48] Wang W., Pei C., Isachenko E., Zhou Y., Wang M., Rahimi G., Liu W., Mallmann P., Isachenko V. (2022). Automatic Evaluation for Bioengineering of Human Artificial Ovary: A Model for Fertility Preservation for Prepubertal Female Patients with a Malignant Tumor. Int. J. Mol. Sci..

[49] Fortune J.E. (2003). The early stages of follicular development: Activation of primordial follicles and growth of preantral follicles. Anim. Reprod. Sci..

[50] Gougeon A. (1996). Regulation of ovarian follicular development in primates: Facts and hypotheses. Endocr. Rev..

